# The beneficial effects of *Moringa oleifera* leaf on reproductive performance in mice

**DOI:** 10.1002/fsn3.918

**Published:** 2019-01-22

**Authors:** Bin Zeng, Junyi Luo, Peng Wang, Lin Yang, Ting Chen, Jiajie Sun, Meiying Xie, Meng Li, Haojie Zhang, Jiajian He, Yongliang Zhang, Qianyun Xi

**Affiliations:** ^1^ Guangdong Provincial Key Laboratory of Animal Nutrition Control National Engineering Research Center For Breeding Swine Industry College of Animal Science South China Agricultural University Guangzhou China; ^2^ Guangdong Medical Laboratory Animal Center Foshan China

**Keywords:** Bax, mice, *Moringa oleifera*, reproduction, sperm abnormality

## Abstract

*Moringa oleifera* is a tropical plant with high nutritional and medicinal value. Recent studies have reported its remarkable effects in inflammatory, antioxidative, and anti‐diabetes modulations, but there was no significant report on its role in animal breeding. In this study, we investigated the effects of dietary *Moringa oleifera* leaf (MOL) on reproductive performances in mice. We studied the reproductive performance of mice for six consecutive gestations. Mice fed with 4% MOL diet showed improved litter size, litter birth weight, and litter survivals until weaning age compared to control mice fed with normal diet (*p *<* *0.05). Mice fed with MOL diet did not change weight and organ coefficients. Serum malondialdehyde (MDA) concentrations in both male and female mice were significantly decreased by dietary MOL (*p *<* *0.05), but glutathione peroxidase (GSH‐PX) and superoxide dismutase (SOD) were unchanged. For male, dietary MOL significantly reduced sperm abnormality rate (*p *<* *0.05) and Bcl2‐associated X protein (Bax) expression in testis (*p *<* *0.05), but did not affect serum testosterone and the expression levels of androgen receptor (AR), phosphoglycerate kinase 2 (Pgk2), protamine2 (Prm2), and B cell leukemia/lymphoma 2 (Bcl2) in testis. For female, dietary MOL did not change serum estradiol and the expressions of estrogen receptor beta (ERβ), Bcl2, Bax, and vascular endothelial growth factor receptor (VEGFR) in ovary. In summary, MOL increased litter size and antioxidant ability, reduced the rate of sperm abnormality and the expression of Bax. Therefore, MOL may serve as a functional feed addictive for improving animal reproductive performance.

## 1 INTRODUCTION

*Moringa oleifera* (MO) is commonly known as drumstick tree or horseradish tree originated from Northern India and Africa, which has been investigated for its application in human health (Leone et al., 2016). The leaves of the *Moringa*, the most utilized part of the plant, are characterized as being highly nutritious, including vitamins, carotenoids, protein, iron, and potassium (Verma, Vijayakumar, Mathela, & Rao, 2009). Moringa leaves also contain an abundance of bioactive compounds, principally polyphenols (phenolic acids and flavonoids) and four unique moringa isothiocyanates, with strong biological activities (Waterman et al., 2015). *Moringa oleifera* leaves can be consumed either fresh or cooked, or be stored as dried powder for many months without refrigeration with little loss of nutritional value (Verma et al., 2009). For these reasons, *Moringa oleifera* leaf (MOL) has been used to treat a number of diseases including cardiovascular disease, insulin resistance, hepatic steatosis, and others (Almatrafi et al., 2017).

*Moringa oleifera* leaf has also shown protective activities in spermatogonial cells and mitigates the cell damage of mice injected with cyclophosphamide (Nayak, Honguntikar, et al., 2016). The hexane extract of MOL has been reported to enhance seminiferous tubule, epididymis, testis, and seminal vesicle functions in male mice (Cajuday & Pocsidio, 2010). In addition, Barakat, Khalil, and Al‐Himaidi (2015) reported that MO combined with hormone supplementations improved the rate of maturation of sheep oocytes and could act as a promoter to induce mRNA expression and synthesis of essential proteins for the maturational processes.

Reproduction is an inevitable composition of life which plays an important role in the survival of human race. For efficient livestock production, advanced reproductive technology is crucial (Hayes, Lewin, & Goddard, 2013), and in mammals, food or nutrient is a key factor in regulating reproductive performance. Some natural plants are known as nutraceuticals, which including functional agents and could bring a positive effect for animal reproduction (Allan & Bilkei, 2005; Guroy, Sahin, Mantoglu, & Kayali, 2012).

However, there is little information on whether dietary MOL could improve reproductive performance in animals. Thus, in this study, we investigated the effects of dietary MOL powder on the reproductive parameters, serum hormones, serum antioxidant indicators, and expressions of essential genes in mice, thereby determining its role in animal reproduction. Not only could these results provide a series of significant data, but also enhance and enlighten the knowledge on development of MOL or its bioactive components in the field of animal reproduction.

## 2 MATERIALS AND METHODS

### 2.1 Animal and experimental diets

Thirty male and 30 female NIH Swiss mice at 4 weeks of age were obtained from the Animal Experiment Center of Guangdong Province (permission number: SYXK [Yue] 2016‐0136). The mice were acclimated for 3 days before the experimental period and maintained under a photoperiod of 12/12 hr (day/night) at a temperature of 24°C ± 2°C and relative humidity of 60% ± 10% throughout the experimental period. The mice had free access to food and drinking water. The mice were randomly assigned to the control group (diet without MOL), 4% MOL group (diet supplemented with 4% MOL), or 8% MOL group (diet supplemented with 8% MOL). All the mice were fed with our experiment feed until sacrificed. At age of 60 days, mice (one female and one male) were mated in one mouse cage and reproduced for six consecutive gestations. MOL powder was purchased from Yunnan Province of China. The chemical compositions of the MOL powder are in Table 1. MOL was mixed evenly in diet, and the diets were custom‐made by Guangdong Medical Laboratory Animal Center. The ingredients and chemical compositions of the three diets are shown in Table 2. All experiments were conducted in accordance with “The Instructive Notions with Respect to Caring for Laboratory Animals” issued by the Ministry of Science and Technology of the People's Republic of China.

**Table 1:** Chemical composition of the MOL (Dry matter basis)

Item (g/kg)	MOL
Crude protein	270.4
Crude fat	74.3
Crude fiber	29.5
Ash	79.6
Calcium	15.8
Total phosphorus	6.1
Fe (mg/kg)	202.3
K	17.9
Mg	4.6
Lysine	13.5
Methionine	2.2
Vitamin E	0.5

**Table 2:** Ingredients and chemical composition of the experimental diets

Item	Groups		
	Control	4% MOL	8% MOL
Ingredients (g/kg)			
Corn grain	396.7	377.2	357.7
Bran	130.0	130.0	130.0
Flour	150.0	150.0	150.0
Bean pulp	160.0	140.0	120.0
Soya‐bean oil	23.0	22.5	22.0
Fish meal	60.0	60.0	60.0
Full‐fat soybean	35.0	35.0	35.0
Mountain flour	15.0	15.0	15.0
Calcium hydrophosphate	20.0	20.0	20.0
Premix^a^	10.0	10.0	10.0
Choline	0.3	0.3	0.3
MOL	0.0	40.0	80.0
Total	1,000.0	1,000.0	1,000.0
Chemical composition (g/kg)			
Carbohydrate	560	560	560
Crude protein	185	185	185
Crude fat	46	46	46
Crude fiber	62.5	62.4	62.4
Calcium	12.8	12.8	12.9
Phosphorus	6.9	6.9	6.9

a Premix provided the following per kg of diets: vitamin A 14,000 IU, vitamin D 1,500 IU, vitamin E 120 IU, vitamin K 5.0 mg, vitamin B_1_ 13.0 mg, vitamin B_2_ 12.0 mg, nicotinic acid 60.0 mg, pantothenic acid 24.0 mg, folic acid 6.0 mg, biotin 0.2 mg, Fe 120 mg, Mn 75 mg, Cu 10 mg, Zn 30 mg/kg, I 0.5 mg, and Se 0.15 mg.

### 2.2 Data and sample collection

Litter size, litter birth weight, average birth weight, litter survival until weaning age (21 days), litter wean weight, and average wean weight were recorded for each pair of mated mice. After the seventh successful pregnancy of female mice, all male mice were sacrificed for the examination of sperm abnormality rate, blood samples, and testis tissues. For female mice, in order to exclude the effect of estrus cycle on the experiment, female mice were sacrificed to collect blood samples and ovary tissue on the 14th day after pregnancy. Observable pessus was regarded as a successful pregnancy (Liu et al., 2007; Ren et al., 2012). Pessus is a white mixture of male seminal vesicle secretion and female vaginal secretion after mating in mice. Generally, it is formed at the vaginal orifice of female mice within 2–4 hr after mating, and can remain at the vaginal orifice for 12–24 hr. Testis or ovary tissues were quick‐frozen by liquid nitrogen. Blood samples were centrifuged at 1,000 × *g* for 20 min at 4°C for serum. The serum and tissue samples were stored at −80°C for further analysis.

### 2.3 Sperm abnormality test

Mice sperm abnormality test was performed as described by Wyrobek and Bruce (1975). Mice were killed by cervical dislocation, and their cauda epididymides were removed. Two sperm suspensions were prepared, each from two cauda epididymides by mincing in 2 ml of phosphate buffered physiological saline, pipetting the resulting suspension and filtering it through an 80‐μm synthetic fiber mesh bag to remove tissue fragments. A fraction (30 μl) of each suspension was then pipetted and smeared at a load fragment to be allowed to dry at room temperature. Then, the load fragments were soaked in methyl alcohol for 5 min for fixation, and stained with 1% Eosin Y, and 60 min later, washed with water. The stained samples were again dried at room temperature. For each suspension, 500 sperms were examined at 400‐fold magnifications; a total of 1,000 sperms were thus examined for each mouse. Abnormal sperms include those with no hook, no head, vacuolar head, amorphous, tail fold, double heads, double tails, etc.

### 2.4 Serum analysis

The testosterone and estradiol in the serum were measured using enzyme‐linked immunosorbent assay kits (ELISA, Nanjing Jiancheng Biotechnology Institute, China). Total SOD, MDA, and GSH‐PX in the serum were determined using spectrophotometric kits (Nanjing Jiancheng Biotechnology Institute) according to the manufacturer's instructions.

### 2.5 Gene expression analysis by quantitative RT‐PCR

Total RNA was extracted from testis and ovary tissue using TRIzol reagent (Invitrogen, Carlsbad, CA, USA) according to the manufacturer's instructions. After treatment with DNase I (Takara Bio Inc., Shiga, Japan), total RNA (2 μg) was reverse‐transcribed to cDNA in a final volume of 20 μl using M‐MLV Reverse Transcriptase (Promega, Madison, WI, USA) plus RNase inhibitor (Promega, Shanghai, China) with oligo‐d(T)s as primers. SYBR Green Real‐time q‐PCR Master Mix reagents (Promega), sense and antisense primers were used for real‐time quantitative polymerase chain reaction (PCR). Quantitative real‐time PCR analysis was performed using CFX96 Touch^™^ Optics Module instrument (BIO‐RAD, California, USA), and β‐actin was used as a candidate housekeeping gene. The following primers were designed: β‐actin, F 5′‐GGTCATCACTATTGGC AACGAG‐3′ and R 5′‐GAGGTCTTTACGGATGTCAACG‐3′; AR F 5′‐GGATGGGACTGATGG TATTTG‐3′ and R 5′‐CAGGATGTGGGATTCTTTCTT‐3′; Pgk2 F 5′‐AGCCTGTGCCAACCC AGATAA‐3′ and R 5′‐CGTAGAACTGTGAGCCCGATG‐3′; Prm2 F 5′‐AGACCATGAACGCG AGGAGCA‐3′ and R 5′‐ATGACCGACGCCTCTTGTGGA‐3′; Bcl2 F 5′‐CCCCTGGCATCTT CTCCTTCC‐3′ and R 5′‐GGGTGACATCTCCCTGTTGACG‐3′; Bax F 5′‐CAGGATGCG TCCACCAAGAA‐3′ and R 5′‐GCAAAGTAGAAGAGGGCAACCAC‐3′; ERβ F 5′‐CTGGGTATCATTACGGTGTCTG‐3′ and R 5′‐CGCCGGTTCTTGTCTATGGT‐3′; VEGFR F 5′‐CTCCA CCTTCAAAGTCTCATC‐3′ and R 5′‐CCCACTACCGAAAGCAATA‐3′.

### 2.6 Western blot analysis

The expressions of Bax protein in testis tissues were determined by Western blot analysis. Total protein was extracted from 100 mg testis tissue using 300 μl radio immunoprecipitation assay (RIPA) lysis buffer that contained 1 mM PMSF and protein phosphatase inhibitor complex (Biosino Bio‐Technology and Science Inc., Beijing, China). Total protein concentration was determined using BCA protein assays (Thermo Scientific Technologies, Wilmington, DE, USA). Protein samples (20 μg) were separated by 10% SDS‐PAGE. The PVDF membranes were then incubated with the indicated antibodies, and rabbit anti‐β‐actin (Bioss) and rabbit anti‐Bax (Sangon, Biotech, Shanghai, China) were used. Protein expressions were measured using a FluorChem M Fluorescent Imaging System (ProteinSimple, Santa Clara, CA, USA) and normalized to β‐actin expression.

### 2.7 Statistical analysis

All data are expressed as means ± standard error of the mean (*SEM*). Data were analyzed by one‐way ANOVA of variance (SPSS 17.0, Chicago, IL, USA). *p *<* *0.05 was considered statistically significant.

## 3 RESULTS

### 3.1 Effects of MOL on reproductive performance

We measured the reproductive performance of mice for six consecutive gestations, and found that average birth weight, litter wean weight, and average wean weight were not significantly different among the control and MOL groups (*p *>* *0.05). However, mice fed with 4% MOL diet had higher litter size, litter birth weight, and litter survival than control mice (*p *<* *0.05). Meanwhile, litter size of mice fed with 8% MOL diet (12.30 ± 0.70) is larger than control mice (10.92 ± 0.55; Table 3). All animals involved in the study looked healthy at the end of the study with no adverse effects observable. We measured body weight and organ weight of male mice and pregnant female mice, and results showed that MOL had no significant effects on body weight or organ coefficients (*p *>* *0.05; Tables 4 and 5). These results suggested that MOL can improve the fecundity of mice without harming their health.

**Table 3:** Reproductive performance of mice (six consecutive gestations, *n* = 10)

Item	Groups			*p*‐value
	Control	4% MOL	8% MOL	
Litter size (*n*)	10.92 ± 0.55^b^	12.76 ± 0.48^a^	12.30 ± 0.70^ab^	0.041
Litter birth weight (g)	20.36 ± 0.82^b^	23.68 ± 0.86^a^	22.38 ± 0.96^ab^	0.033
Average birth weight (g)	1.93 ± 0.04	1.87 ± 0.02	1.90 ± 0.05	0.521
Litter survivals (*n*)	10.72 ± 0.53^b^	12.20 ± 0.47^a^	11.92 ± 0.67^ab^	0.048
Litter wean weight (g)	135.98 ± 3.07	142.07 ± 6.87	139.18 ± 5.20	0.736
Average wean weight (g)	13.21 ± 0.39	12.92 ± 0.66	13.05 ± 0.59	0.853

^ab^Different superscripts within a row represent significant differences (*p *<* *0.05).

**Table 4:** Weight and organ coefficients of male mice (*n* = 10)

Item	Groups			*p*‐value
	Control	4% MOL	8% MOL	
Weight (g)	44.82 ± 1.17	44.73 ± 1.05	43.93 ± 0.97	0.733
Testis coefficient (%)	0.53 ± 0.03	0.54 ± 0.03	0.53 ± 0.03	0.853
Heart coefficient (%)	0.43 ± 0.01	0.44 ± 0.01	0.44 ± 0.02	0.673
Liver coefficient (%)	4.32 ± 0.14	4.40 ± 0.13	4.46 ± 0.16	0.796
Spleen coefficient (%)	0.29 ± 0.01	0.29 ± 0.01	0.28 ± 0.01	0.717
Lung coefficient (%)	0.51 ± 0.02	0.49 ± 0.01	0.50 ± 0.02	0.718
Kidney coefficient (%)	1.29 ± 0.06	1.34 ± 0.02	1.32 ± 0.06	0.606

**Table 5:** Weight and organ coefficients of pregnant female mice (*n* = 10)

Item	Groups			*p*‐value
	Control	4% MOL	8% MOL	
Weight (g)	59.54 ± 1.54	58.77 ± 1.36	58.42 ± 1.46	0.812
Ovary coefficient (%)	0.03 ± 0.001	0.03 ± 0.002	0.03 ± 0.001	0.786
Heart coefficient (%)	0.36 ± 0.02	0.37 ± 0.02	0.36 ± 0.02	0.611
Liver coefficient (%)	4.16 ± 0.18	4.11 ± 0.16	4.19 ± 0.17	0.677
Spleen coefficient (%)	0.24 ± 0.04	0.25 ± 0.05	0.23 ± 0.03	0.521
Lung coefficient (%)	0.43 ± 0.02	0.46 ± 0.03	0.44 ± 0.02	0.533
Kidney coefficient (%)	1.02 ± 0.04	1.08 ± 0.03	1.05 ± 0.03	0.764

### 3.2 Effects of MOL on serum hormone and antioxidant indexes

*Moringa oleifera* leaf had no significant effects on the serum testosterone concentration in male mice and serum estradiol concentration in female mice (Tables 6 and 7, *p* > 0.05). Serum antioxidant indexes were measured, and the results showed that serum glutathione peroxidase (GSH‐PX) and superoxide dismutase (SOD) in male or female mice were unchanged (*p *>* *0.05). But male and female mice fed with MOL diets had lower serum malondialdehyde (MDA) than control mice (Tables 6 and 7), which indicated that MOL could, in some extent, play an anti‐oxidative role in mice.

**Table 6:** Serum testosterone and antioxidant indexes of male mice of fed MOL (*n* = 8)

Item	Groups			*p*‐value
	Control	4% MOL	8% MOL	
Testosterone (nmol/L)	33.65 ± 2.62	33.08 ± 2.38	33.31 ± 3.10	0.989
Glutathione peroxidase (U)	1,042.50 ± 66.04	1,143.67 ± 73.85	1,133.57 ± 53.74	0.481
Superoxide dismutase (U/ml)	57.36 ± 1.11	59.75 ± 1.32	59.98 ± 1.05	0.185
Malondialdehyde (nmol/ml)	8.00 ± 0.53^a^	6.33 ± 0.50^b^	6.14 ± 0.64^b^	0.034

^ab^Different superscripts within a row represent significant differences (*p *<* *0.05).

**Table 7:** Serum estradiol and antioxidant indexes of female mice of fed MOL (*n* = 8)

Item	Groups			*p*‐value
	Control	4% MOL	8% MOL	
Estradiol (ng/L)	6,569.25 ± 348.02	6,558.38 ± 385.61	6,681.12 ± 486.32	0.973
Glutathione peroxidase (U)	1,011.27 ± 27.13	1,084.83 ± 65.21	1,072.04 ± 94.92	0.658
Superoxide dismutase (U/ml)	59.74 ± 1.26	60.81 ± 0.77	62.48 ± 0.60	0.089
Malondialdehyde (nmol/ml)	8.87 ± 0.54^a^	7.32 ± 0.49^ab^	7.01 ± 0.55^b^	0.047

^ab^Different superscripts within a row represent significant differences (*p *<* *0.05).

### 3.3 Effects of dietary MOL on sperm abnormality rate and expressions of genes in testis for male mice

**Figure 1:**
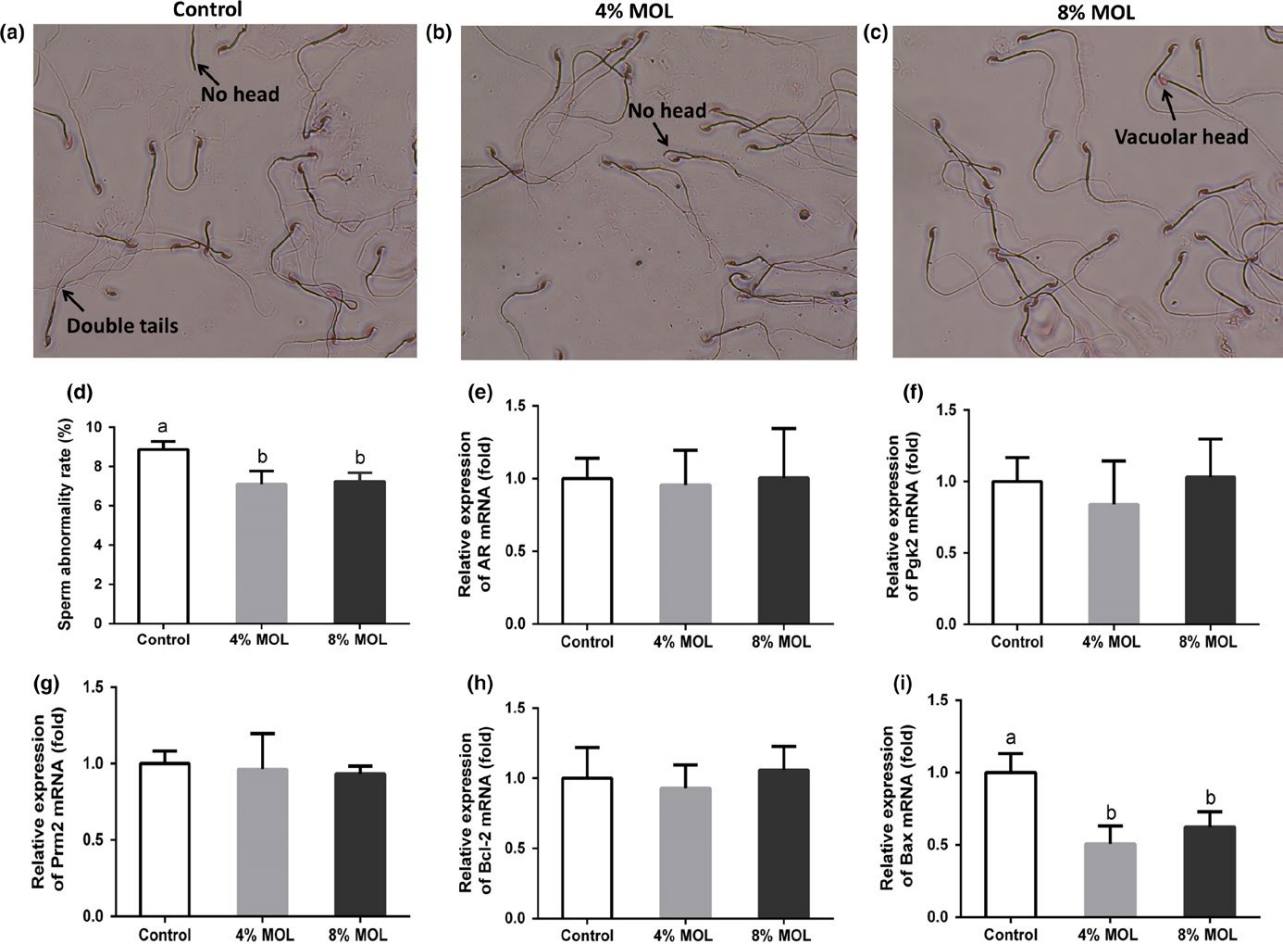
Microscopic observation (400×) of sperm morphology for control (a), 4% MOL (b) and 8% MOL (c). The sperm abnormality rates of male mice (d). Relative mRNA levels of AR (e), Pgk2 (f), Prm2 (g),Bcl‐2 (h), and Bax (i) by using quantitative PCR in testis tissue of male mice. Data are presented as mean ± *SEM*,* n* = 8. Results are normalized to β‐actin. Different superscripts “a”/“b” represent significant differences between groups (*p *<* *0.05). Control: diet without MOL, 4% MOL: diet supplemented with 4% MOL, and 8% MOL: diet supplemented with 8% MOL

**Figure 2:**
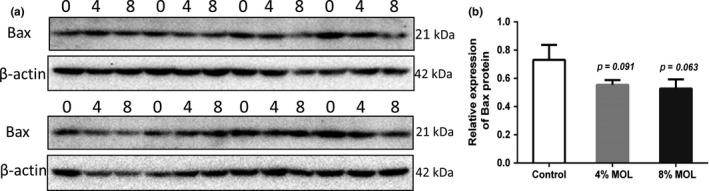
(a) The protein levels of Bax measured by Western blot. “0” means diets with no MOL, “4” means diets supplemented with 4% MOL, and “8” means diets supplemented with 8% MOL. (b) The statistical analyses results of the Western blot of the protein level of Bax. Data are presented as mean ± *SEM*,* n* = 8. Results are normalized to β‐actin

The sperm abnormality rate of male mice was also determined to examine the effect of MOL on semen quality. The microscopic observation of sperm morphology is shown in Figure 1A–C. Interestingly, MOL‐fed groups had lower sperm abnormality rate than control group (Figure 1D). These observations demonstrated that MOL could improve semen quality of mice. We determined the expressions of genes related to spermatogenesis in testis tissue. The expression levels of androgen receptor (AR), phosphoglycerate kinase2 (Pgk2), protamine2 (Prm2), and B cell leukemia/lymphoma 2 (Bcl2) in testis remained unchanged after feeding dietary MOL (Figure 1E–G). Importantly, Bcl2‐associated X protein (Bax) was reduced in the MOL groups male mice relative to the control group (Figure 1I), which was further confirmed by Western blot (Figure 2A,B). These results indicated that MOL may boost mice reproduction by improving sperm quality and inhibiting testicular apoptosis.

### 3.4 Effects of MOL on gene expression in ovary for mice

**Figure 3:**
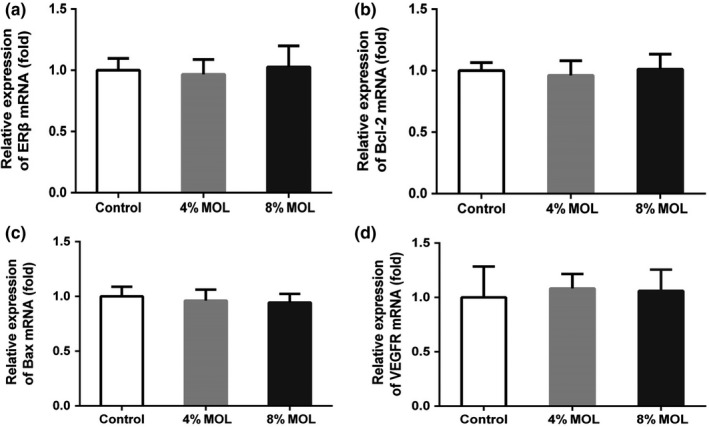
Relative mRNA levels of ERβ (A), Bcl‐2 (B), Bax (C), and VEGFR (D) using quantitative PCR in ovary tissue of female mice. Data are presented as mean ± *SEM*,* n* = 8. Results are normalized to β‐actin. Control: diet without MOL, 4% MOL: diet supplemented with 4% MOL, and 8% MOL: diet supplemented with 8% MOL

In female mice, mRNA levels of estrogen receptor beta (ERβ), Bcl2, Bax, and vascular endothelial growth factor receptor (VEGFR) in ovary tissue were not significantly different among groups (Figure 3A–D). These results indicated that MOL did not change mRNA expressions of reproductive related genes in ovary.

## 4 DISCUSSION

In this study, MOL was adopted for the first time to identify the effect on long‐term (six consecutive gestations) reproductive performance of mice. Our results indicated that dietary MOL was able to improve litter size, litter birth weight, and litter survival. Litter size and litter survival are important indicators for reproduction performance (Rothschild, 1996). A similar study carried out by Odeyinka, Oyedele, Adeleke, and Odedire (2008) showed that rabbits receiving 100% Moringa diet had higher litter size, litter weight at birth and litter weight at weaning than those receiving 100% Centrosema. Additionally, it was also reported that in combination with hormone supplementation, MOL improved the rate of maturation of sheep oocytes and could act as a promoter to induce mRNA expressions and synthesis of essential proteins for the maturational processes (Barakat et al., 2015). They concluded that improvement of fertility by MOL administration might be due to its chemical compositions in the leaves as an excellent source of nutrients. Moreover, MOL contains beta‐carotene and other strong antioxidative phytochemicals (kaempferol, quercetin, rutin, and caffeoylquinic acids), essential antioxidative micronutrients (selenium and zinc as explained) and antioxidative vitamins (C, E, and A), that have regulative effects in fertility performance (Jaiswal, Rai, Kumar, Mehta, & Watal, 2009; Vongsak, Sithisarn, & Gritsanapan, 2014). In agreement with the above findings, the current study showed an improvement in reproduction performance of mice fed with MOL diet. Moreover, mice fed with 8% MOL diet did not work as well as the mice fed with 4% MOL. A possible reason is that 8% MOL feed contain higher alkaloids. (Sahakitpichan, Mahidol, Disadee, Ruchirawat, & Kanchanapoom, 2011).

In the present study, the body weight and organ coefficient of the MOL‐treated mice remained unchanged, which demonstrated that MOL dose is safe in animal application. The levels of serum hormones play an important role for the sexual capacity of mammals. In present study, MOL had no significant effects on the serum testosterone concentration in male nor on the serum estradiol concentration in female mice. Similar results have been reported by Cajuday and Pocsidio (2010) who fed male mice with a hexane extract of MOL.

Malondialdehyde is considered a presumptive biomarker for lipid peroxidation in live organisms (Mateos, Lecumberri, Ramos, Goya, & Bravo, 2005). In our study, dietary MOL decreased serum MDA. Oparinde and Atiba (2014) also observed that serum MDA was significantly lower in rats fed with *Moringa oleifera* than those with normal diet. Additionally, *Moringa oleifera* leaf and fruit extracts were found to reduce MDA levels both in vitro and in vivo (Luqman, Srivastava, Kumar, KumarMaurya, & Chanda, 2012). Previous studies demonstrated that the MOL possessed potent antioxidant properties due to its high contents of phenolic compounds and isothiocyanate (Tumer, Rojas‐Silva, Poulev, Raskin, & Waterman, 2015; Verma et al., 2009).

An important discovery in our study is that MOL‐fed groups had lower sperm abnormality rates than the control group. Sperm abnormalities have long been associated with male infertility and sterility in most species (Saacke, 2001). Up to date, there is little published data concerning the effect of MOL on sperm abnormalities in animals. Cajuday and Pocsidio (2010) reported that male mice administered with the hexane extract of MOL showed increased spermatids in their seminiferous tubules by testicular histology. Additionally, *Moringa oleifera* leaf extracts could ameliorate electromagnetic radiation or cyclophosphamide induced sperm damage in rat and mice (Bin‐Meferij & El‐Kott, 2015; Nayak, Vadinkar, et al., 2016). The exact mechanism for the decrease in the frequency of abnormal sperm is not clear. It was suggested that lower sperm abnormality resulted from lower chromosome abnormality and less minor alterations in testicular DNA and point mutation.

It is well known that mouse reproduction is regulated by a few crucial genes. AR and ERβ are essential for normal fertility efficiency of mice (Krege et al., 1998; Shiina et al., 2006). Pgk2 and Prm2 are crucial for maintaining the sperm motility, sperm chromatin integrity, and male fertility (Cho et al., 2003; Danshina et al., 2010). In the present study, the gene expressions of AR, Pgk2, and Prm2 in testis and ERβ in ovary seemed unchanged after feeding dietary MOL. The possible reason for this result is that all group mice are under normal and health condition and maintained normal physiological status while the functions of these key genes are to maintain normal reproduction processes. This result also demonstrated that MOL had no harmful effect on animal reproduction physiology. We also determined the expressions of Bcl‐2 and Bax, which are related to cell apoptosis. Interestingly, dietary MOL decreased the expression of Bax in testis tissue, proved both by qRT‐PCR and Western blot. A previous study demonstrated Bcl‐2 protein was an inhibitor of apoptosis, while Bax was an accelerator (Misao et al., 1996). The pre‐set ratio of Bcl‐2/Bax seemed to determine the survival or death of cells following an apoptotic stimulus (Albamonte et al., 2008). Our results suggested that MOL may suppress testis cell apoptosis by down‐regulating Bax expression. Meanwhile, it has been reported that Bcl‐2/Bax was closely connected with antioxidative pathway (Yang, Wang, Xie, Sun, & Wang, 2010). Therefore, the antioxidative phytochemicals in MOL may change the expression of Bax. However, the exact mechanism has not been explored yet.

## 5 CONCLUSION

In summary, we have investigated the beneficial effects of MOL on animal reproduction. Our studies showed that dietary MOL improved reproductive performances of mice. Dietary MOL decreased serum MDA in both male and female mice, and more importantly, MOL reduced the rate of sperm abnormality in male, and the expression of Bax. Our findings provided a basis for the further understanding of MOL supplementation and animal reproduction. Further studies are necessary to search for the active constituents of MOL and its specific mechanisms.

## ETHICAL STATEMENT

The authors declare that there is no conflict of interests. All experiments were conducted in accordance with “The Instructive Notions with Respect to Caring for Laboratory Animals” issued by the Ministry of Science and Technology of the People's Republic of China. The study's protocols and procedures were ethically reviewed and approved by South China Agricultural University animal experiment ethics review committee.
